# Humanin gene expression in fibroblast of Down syndrome subjects

**DOI:** 10.7150/ijms.39145

**Published:** 2020-01-18

**Authors:** Michele Salemi, Federico Ridolfo, Maria Grazia Salluzzo, Rossella Cannarrella, Mariaconcetta Giambirtone, Salvatore Caniglia, Cataldo Tirolo, Raffaele Ferri, Corrado Romano

**Affiliations:** 1 Oasi Research Institute-IRCCS, Troina (EN), Italy.; 2 UOSD of Clinical Pathology, ASUR Marche -AV2, Hospital of Senigallia, Senigallia, Italy.; 3 Department of Clinical and Experimental Medicine, University of Catania, Catania, Italy.

**Keywords:** Down syndrome, Intellectual Disability, Expression, Humanin gene, Immunofluorescence, qRT-PCR.

## Abstract

Down syndrome (DS) is characterized by trisomy of chromosome 21 and peculiar phenotype. Humanin (HN) is a mitochondrial short 24-residue polypeptide whit anti-apoptotic and neuroprotective effects. In this study we evaluated HN protein expression and HN mRNA levels in cultured fibroblasts from DS patients and normal controls. Our results obtained by immunocytochemistry, western-blot and qRT-PCR analysis show a significant HN up-regulation in DS patients. These results confirm previous studies and suggest a role for HN may in the DS phenotype.

## Introduction

Down syndrome (DS), which is associated with the trisomy of chromosome 21, is the leading genetic cause of intellectual disability (ID) 1. Trisomy 21 is due to defective chromosome disjunction at the first or second meiotic division 1. Several biomarkers associated with aging have been identified in nervous system of DS persons 2. Humanin (HN) plasma levels have been recently positively correlated with age in humans 3 (Conte et al., 2018). Interestingly, higher HN serum levels have been reported in DS subjects compared to normal control (NC) brothers 3. Overall, this supports clinical observations that subjects with DS have an accelerated aging phenotype.

HN is a mitochondrial short 24-residue polypeptide expressed in response to mitochondrial stress 4. It has anti-apoptotic and neuroprotective effects. The aim of the present study was to evaluate HN protein levels and HM mRNA expression in fibroblasts from DS subjects, compared to those of fibroblasts from NC subjects.

## Materials and Methods

### Subjects

All subjects were recruited after obtaining family/tutor and/or personal consent at the Oasi Research Institute - IRCCS, Troina, Italy, a specialized center for ID and brain aging. Human fibroblasts were obtained by oral biopsy of periodontal gingival tissue from 19 unrelated NC subjects (eight men and 11 women, age range 18-42 years) and 19 DS subjects (eight men and 11 women, age range 20-42 years). The diagnosis of Trisomy 21 was confirmed by karyotyping. Patients and controls were recruited in a case/control design. The study was approved by the Oasi Research Institute Ethical Committee (2017/05/31/CE-IRCCS-OASI/9 of 3 June 2017).

### Biological material

Human gingival fibroblasts were isolated from explants of human gingiva and cultured in Dulbecco's modified Eagle's medium as described by Salemi et al. (2012) 5 .

### RNA extraction and qRT-PCR

RNA extraction and retro-transcription were performed as described by Salemi et al. (2012) 5. The amplified transcripts were quantified using the comparative 2^-ΔΔCt^ method 6, matching by age and sex every DS subject with the respective NC subject.

Custom TaqMan Humanin Gene expression, was normalized to *GAPDH* levels. Custom TaqMan Gene expression Assay for Humanin (forward primer: tggctccacgagggttca; reverse primer: tatgcccgcctcttcacg and and probe sequence: tttaaccagtgaaattgacc) and the reference gene glyceraldehyde-3-phosphate dehydrogenase (GAPDH) TaqMan assays (Assay ID: Hs99999905_m1) were obtained from Applied Biosystems (Carlsbad, CA, USA). mRNA levels were calculated using the Light Cycler 1.5 software supplied with the Light Cycler 480.

### Immunofluorescence

Samples were fixed with 4% formalin for 30 min at 4 °C and post-fixed with 70% ethanol for 24 hours at 20 °C; 0.2 ml of suspension containing 20×10^6^ fibroblasts/ml in culture medium were incubated for 1 hour at room temperature with the primary anti-HN antibody (1:200), a rabbit polyclonal antibody raised against HN protein (Thermo Fischer Scientific, Rockford, IL61105, USA). The secondary antibody used was FITC-labelled goat anti-rabbit IgG (1:30) (Sigma-Aldrich Corp., St Louis, MO, USA). Nuclei were counter-stained with 100 ng/ml 4ʹ,6-diamidino-2-phenylindole (DAPI) (Cytocell, Banbury, UK). Slides were observed and cells were visually scored at 200× and 400× magnification. Immunostaining was examined using a fluorescent microscope BX-51 (Olympus,Jappan). Of all cases and controls at least 200 cells were examined. Cells examined were classified with the following scoring: high level positivity ++, slight positivity + -, negative -. Levels ++ and + - were considered positive for statistics.

### Western blot analysis

Fibroblast proteins were quantified using the BCA protein determination method (Bio-Rad, Hercules, CA, USA), solubilized in Laemmli buffer, at a concentration of about 1-2×102 fibroblasts/ml in the presence of 5% β-mercaptoethanol at 100°C, electrophoresed on 15% polyacrylamide-SDS gel and electro-blotted onto nitrocellulose membrane (Bio-Rad, Philadelphia, PA, USA) for 2 h at 0.24 mA/cm2. Both HN and β-Actin migrated to the samepolyacrylamide-SDS gel.

Protein bands were detected on the membrane using anti-HN primary antibody (Humanin Polyconal Antibody, PA1 41326; Thermo Fischer Scientific, Rockford, IL61105, USA) and in parallel with anti- β-Actin (Sigma Life Actin primary antibody, St. Louis, USA). A goat anti-rabbit antibody-HRP conjugate (Goat-Anti- Rabbit secondary Antibody, #31460, Thermo Fisher Scientific Inc., Rockford, IL, USA) was used as the secondary antibody.

Quantitative analysis of photographed bands was carried out with ImageJ software. Density value of HN protein bands was quantified in terms of pixels and it was normalized to β-Actin value protein bands.

### Statistical analysis

Distribution of HN mRNA levels was analyzed using the Shapiro-Wilk's test; Wilcox on rank-sum test, *t*-test and bivariate linear regression analysis were used for inferential statistical analysis. The significance level was set at a *p* value <0.05. The Graph Pad Prism 5 software was used for statistical analysis.

## Results

### qRT-PCR

**Figure 1:**
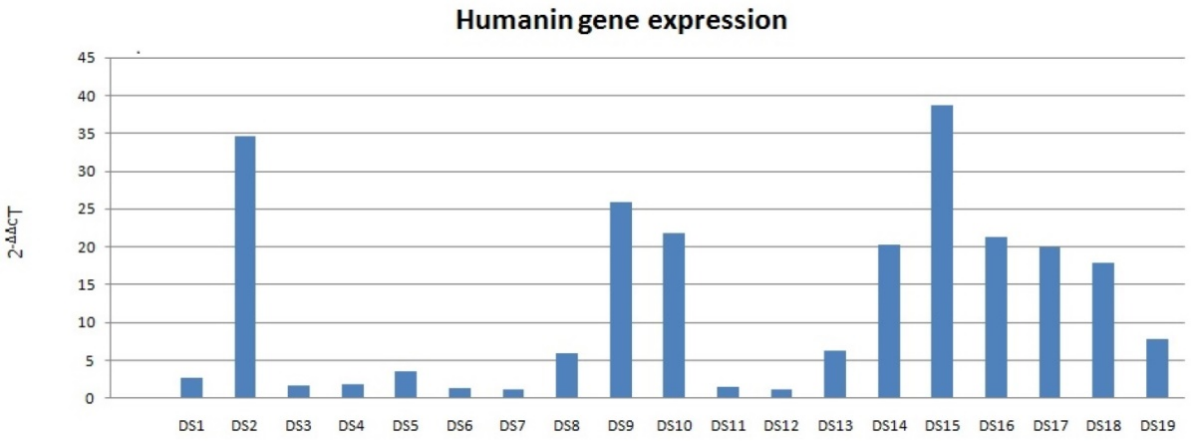
Humanin mRNA expression in DS. Data obtained by qRT-PCR.

Increased HN mRNA levels were found in all 19 DS samples; among these, 13 DS samples had a mRNA value higher than the double of the coupled NC (Fig. 1). HN mRNA levels were not normally distributed (p <0.01). Therefore, the Wilcoxon rank-sum test was used for inferential statistical analysis, showing significantly higher DS HN mRNA levels compared to coupled NCs (p <0.01).

**Figure 2:**
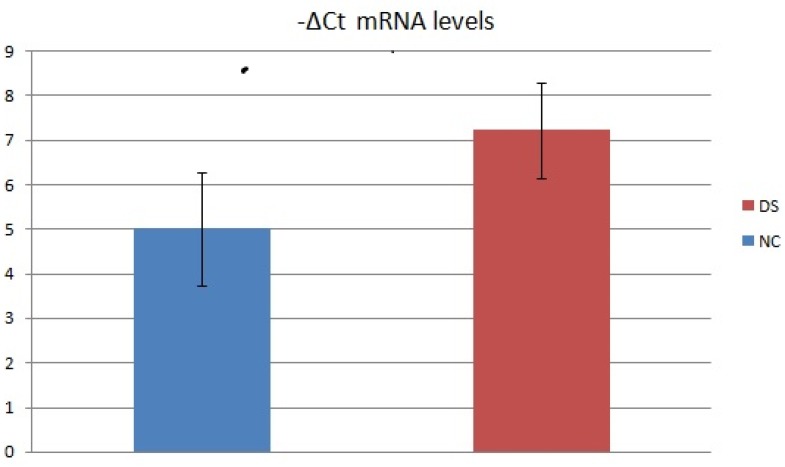
Average -ΔCt mRNA levels value of two DS and NC groups.

We also assessed the mRNA levels of 2 subject groups by evaluating the relative expression value in terms of -ΔCt (Fig.2). In this analysis, the mean mRNA levels of NC was 5,00 (SD=6,58; CV=1,28; IC 95% = 1,83 - 8,17). The mean mRNA levels of DS subjects were 7,22 (SD=7,9; CV=1,06; IC 95% = 3,42 - 11,03). Inferential statistical analysis revealed significant difference between the two groups (*p*<0.05). No significant statistical effect was found for gender (p >0.05) and no linear correlation was found with age (p >0.05).

### Immunofluorescence

**Figure 3:**
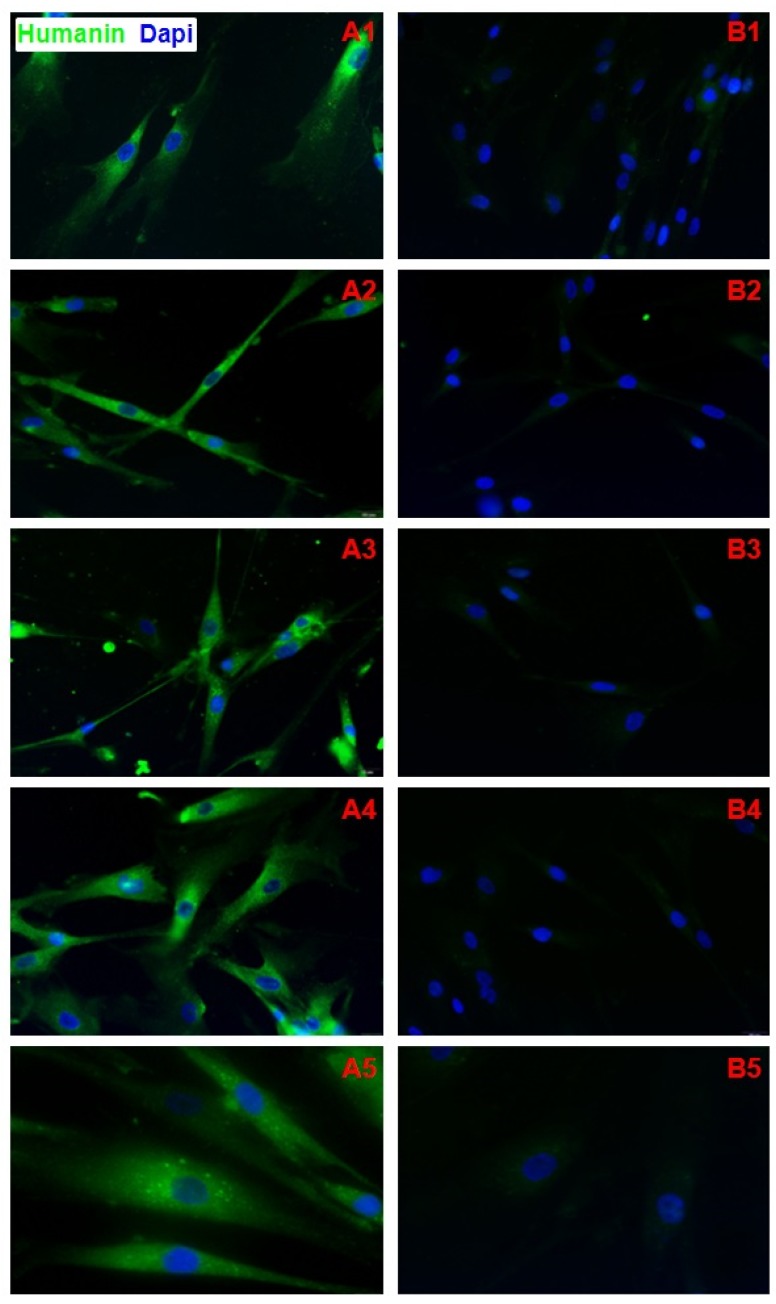
A1-A5) Immunofluorescence of fibroblasts obtained from oral biopsy of periodontal gingival tissue in DS subject, Humanin protein green fluorescence, nuclei were counter-stained in blue with 100 ng/mL 4,6-diamidino-2-phenylindole (DAPI); B1-B5) Immunofluorescence of fibroblasts obtained from oral biopsy of periodontal gingival tissue in normal subject, Humanin protein green fluorescence, nuclei were counter-stained in blue with 100 ng/mL 4,6-diamidino-2-phenylindole (DAPI).

HN protein was observed in both NC and DS fibroblast cytoplasm (Fig. 3A1-5). No significant HN nuclear signal was detected in DS and NC fibroblasts (Fig. 3B1-5). CN samples showed a positive cells rate of 8.13 on 100 cells analyzed ( DS=2,99; CV=0,35; IC 95%=6,69 - 9,57); DS samples showed a positive cells rate of 97.58 on 100 cells analyzed ( DS=0,77; CV=0,008; IC 95%=97,21 - 97,88). Difference in expression appears to be statistically significant (*p*<0,0001). No significant statistical effect was found for gender (p >0.05) and no linear correlation was found with age (p >0.05).

### Western blot analysis

**Figure 4:**
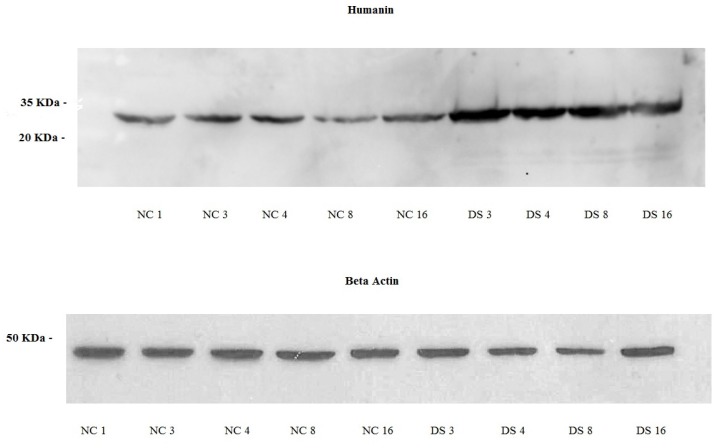
Western blot of normal fibroblast cell lines NC1, NC3, NC4, NC8 and NC16 (Controls subjects) and a DS3, DS4, DS8 and DS16 cell lines (DS subjects), Humanin protein approximately 30 kDa, β-Actin protein approximately 45 kDa.

Western blot analysis revealed the presence of HN protein in fibroblasts of both DS subjects and controls. The protein had a molecular mass of approximately 30 kDa (Fig.4). DS samples showed a significantly higher overage expression level (1,24; SD=0,2; CV=0,14; IC 95%=0,92 - 1,55) compared to NT (0,53; SD=0,25; CV=0,42; IC 95%=0,22 - 0,85) (p<0,01). It did not differed for sex (p>0,05) or age (p>0,05).

## Discussion

Conte et al. (2018) 3 analyzed HN, FGF21, and GDF15 plasma levels in 693 subjects (whose age ranged from 21 to 113 years). They resulted to be increased in elderly, being particularly high in the centenarians. These molecules are associated with pathological physical and biochemical parameters (such as handgrip strength, insulin sensitivity and elevated triglycerides) 3. It cannot be excluded a role for high cellular HN levels to compensate for the high production of free radicals.

DS is associated with several diseases, such as metabolic syndrome, dyslipidemia and hyperinsulinemia 7,8. The presence of mitochondrial dysfunction could elicit the production of effective stress responses (including HN production). We have already highlighted a down-regulation of various mitochondrial subunits in fibroblasts of DS subjects 5. This do not relate with the trend of HN expression described in the current study. Our data showed a greater HN protein and mRNA expression in DS subjects.

Our findings strengthen the results by Conte et al. (2018) 3. Indeed, we showed that a difference between DS subjects and coupled non-brother NC does exist.

This may possible reflect the potential role of HN in the various aspects of the phenotype of subjects with DS, such as premature aging mechanisms and several autoimmune factors related to DS syndrome. Indeed, the increased expression of HN might represent the response to such events.

Qin et al. (2018) 9 have highlighted that exogenous HN treatment attenuated myocardial fibrosis and apoptosis in aged mice, suggesting a role for the mitochondria-derived peptide HN in cardioprotection. The study by Yen et al. (2018) 10 addressed to HN a role in neuroprotection against cognitive aging in humans. In addition, Solanki et al. (2018) 11 proposed a potential application of a HN derivative for treating age-related macular degeneration (AMD) since AGA-HNG may represent a promising therapeutic option for AMD. On these basis, HM may be a therapeutic target in aging-related diseases.

People with DS are at increased risk of Alzheimer's disease and the 50% of patients whit DS develop dementia in their fifth or sixth decade of life 12. Gao-Shang Chai et al. (2014) 13 have found that HN attenuates: the Amyloid β-peptide induced memory deficits, tau phosphorylation, dendritic degeneration, neuronal apoptosis and neuronal dysfunction; then could ameliorate the senile dementia present in individuals with Alzheimer's disease 13. Bik-Multanowski et al. (2015) have highlighted that the expression of MTRNR2L12 (a humanin isoform) might be a new, easy-to-measure blood marker of severe cognitive disability and, possibly, of early dementia in patients with Down syndrome 13.

In our study no significant statistical effect was found for gender (p >0.05) and for age (p >0.05). This makes us suppose, that high levels of HN, in our casuistry, are related only to the subject status with DS. In any case, a study with a wider range of cases, where a subset of subjects with Alzhemir's disease can be identified is desirable as soon as possible.

In conclusion, our results confirm previous findings (Conte et al. 2018) 3 expanding the current knowledge. They could serve as a basis for future studies on possible therapeutic use of HN to treat DS-associated phenotypic features, especially if implemented during the early developmental period.
